# Effects of country animosity of angry Koreans on Japan: A focus on export regulation on Korea

**DOI:** 10.3389/fpsyg.2022.961454

**Published:** 2022-10-25

**Authors:** Lili Sun, Jong-Woo Jun

**Affiliations:** ^1^Department of Basic Teaching, Hebei Academy of Fine Arts, Shijiazhuang, China; ^2^Department of Communication, Dankook University, Yongin, South Korea

**Keywords:** ethnic identity, media uses, animosity, government trust, boycott visit intention

## Abstract

Nowadays, Korea and Japan are in conflict arising from export restrictions launched by Japan on Korea, which have provoked a boycott of Japanese products in Korea, and even tourism to Japan. Animosity performs a momentous role in the context of crisis management communication. Hence, this article aims to investigate factors impacting boycott intention to visit Japan, with economic animosity being a principal mediating variable, whose antecedents and consequences have been probed into. A total of 333 respondents' survey data were collected and analyzed *via* SEM for the verification of research hypotheses. The findings manifest that ethnic identity engenders significant direct positive bearings upon economic animosity and boycott news, and boycott news significantly positively affects economic animosity; boycott news serves as the mediating role between ethnic identity and economic animosity. Additionally, the outcomes denote that economic animosity exerts a significant positive impact on boycott visit intention, economic animosity negatively affects Japanese government trust, and Japanese government trust negatively bears upon boycott visit intention; Japanese government trust mediates between economic animosity and boycott intention to visit Japan. Consequently, the research makes contributions to furnishing empirical evidence for influencing factors of boycott visit intention and enriching the literature on the antecedents and consequences of animosity.

## Introduction

Globalization is in crisis. A trade war is also waged in the interests of the country. The trade conflict, which was triggered by the U.S.A. imposing sanctions on Chinese IT company Huawei, is having a negative impact on the global economy. Additionally, as Japan enforces export restrictions on Korea, Asia is entering a serious conflict. In the reality of nationalism around the world, the understanding of conflicts between countries now requires a close examination not only on diplomatic matters but also on the economic level.

Currently, Korea and Japan are in conflict. Conflicts that began with Japan's export restrictions have spread to the boycott of Japanese products. The boycott has influenced Japanese products as well as travel to Japan. Moreover, when it was revealed that it was made in Japan, like Uniqlo and Japanese beer, it was quite a hit in Korea. With the outbreak of COVID-19 all over the world, the physical boycott of Japanese-made products has eased a little, but negative perceptions of each other between the two countries have reached their peak. Although the Japanese government's leadership has changed, the current government maintains the diplomatic strategy of the Abe regime, and therefore it seems to take time to improve relations with Korea.

The countries that are the target of boycotts can be severely damaged not only economically, but also in terms of their national images. The recent trade conflict between America and China has resulted in the U.S.A.'s restrictions on Chinese-made products. The negative impact on products from these conflicts can be explained by animosity toward the countries (Klein et al., 1998). The establishment of a positive national image is vital to increase national competitiveness, but strategically managing national hostility in the context of crisis management communication is also significant for national brands.

Hence, this study aimed at exploring the factors affecting the boycott intention to visit Japan with economic animosity being a major mediating variable. To attain the research purpose, concrete research objectives were drawn up as follows: (1) To probe into the influencing factors of boycott intention to visit Japan for the sake of the comprehension of the impacts of animosity and government trust on boycott visit intention. (2) To examine antecedents and consequences of economic animosity, with economic animosity being a main mediating variable, ethnic identity, and boycott news use being antecedents, and Japanese government trust and boycott visit intention being consequences to understand the bearings of ethnic identity and boycott news on economic animosity, along with the effects of economic animosity on Japanese government trust and boycott intention to visit Japan.

## Conceptual framework and research model

### Ethnic identity

Identity as a guideline for individual behavior, people ought to engage with others, think and act in a consistent manner within their group, because “there is uniformity in thought and action in being a group member” (Burke and Stets, 2009, p. 118). Ethnic identity means the degree of an individual's connection to the original culture together with adherence to traditional values (Olmedo, 1979), so ethnic identity underlines an individual's affiliation with an ethnic group (Pires et al., 2003). Ethnic identity involves a sense of belonging to one's own group, clear comprehension of membership's meaning, positive attitudes toward one's own group, familiarity with the group's culture and history, and participation in its practices (Phinney et al., 1994). In the research of Jun et al. (2021), the ethnic identity of Koreans is viewed as a cultural orientation toward Korean identity. Also, the ethnic identity of Koreans can be summarized as a community spirit that values more community interests than individual ones (Lee and Park, 2017).

Ethnic identity has been inspected for all the groups from three dimensions, that is, self-identification, belonging sense along with attitudes toward an ethnic group of one's own, as well as ethnic practices and behaviors (Phinney, 1992). Concretely speaking, self-identification signifies an ethnic label utilized for individuals (Phinney, 1992), which is an essential prerequisite for ethnic identity. Individuals who come from blended backgrounds may make choices of their ethnic labels and may gain identification with several ethnic groups simultaneously. The second one denotes individuals' integration into social collectivity through the assumption of given roles (Pollini, 2005). As the final dimension, ethnic behaviors and practices lay stress on one's participation in cultural and social activities within a group (Phinney, 1992). Moreover, the intensity of ethnic identity can change over time (Pires et al., 2003). In other words, ethnic identity is dynamic, involving adaptation, transformation, and/or evolution (Lindridge, 2010), which was utilized for the assessment of individuals' social and psychological transformations (Hirschman, 1981; Webster, 1991; Laroche et al., 2005). In addition, identification with an ethnic group acts as individual behavior's point of reference, and ethnic identity can also influence consumers' unfavorable feelings toward foreign countries (El Banna et al., 2018).

### Animosity

Animosity means both open hostility and ideas of enmity, ill will, along with displeasure (Shimp et al., 2004). Besides, animosity represents the ingrained strong emotions triggered by prior or ongoing economic, military, or political events, which can be composed of four types, that is, stable, situational, personal, as well as national animosity (Ang et al., 2004). Stable animosity denotes unfavorable emotions stemming from historical background, for instance, prior military or economic relationships between nations, whereas situational animosity signifies negative emotions associated with a particular situation, e.g., a present economic and/or political event. Jung et al. (2002) contend that stable animosity is inclined to accumulate over time, while situational animosity originates from a particular episode or event and harbors temporary nature. Personal animosity can be generated from an individual's negative personal experiences with (people of) one or more foreign countries, whereas national animosity, namely, animosity toward a country, arises from memories of and perspectives on a foreign country's treatment of the home country.

Furthermore, animosity is multidimensional (Amine et al., 2005), among which six have been summarized (Yu et al., 2020), i.e., war, economic, political, religious, cultural, and people-related/social animosity. War animosity along with economic animosity is determining factor of general animosity (Klein et al., 1998). War animosity indicates hostility engendered by war aggression or military acts launched by a country against another (Klein et al., 1998); war animosity is generally stable in essence (Mrad et al., 2013). Economic animosity may be produced by economic disputes between two countries (Klein et al., 1998), and economic animosity is mainly situational (Mrad et al., 2013). Situational economic animosity originates from feelings of a country's population about economic aggression or domination against a hostile country (Nijssen and Douglas, 2004), and is associated with temporary economic events (Jung et al., 2002), e.g., trade disputes along with economic arguments (Mainolfi, 2021). Therefore, Korean people's animosity resulting from Japan's export restrictions to Korea belongs to situational economic animosity, which will be explored in this study.

At times, consumers reject the purchase of foreign-made products because of their animosity toward a certain country (Mrad et al., 2013). For instance, a large number of Chinese consumers have avoided purchasing Japanese products as a result of the 1937 Nanjing Massacre (Klein et al., 1998). Although the massacre occurred over half a century ago, numerous Chinese consumers avoid buying Japanese products (Mrad et al., 2013).

Three antecedents were found to have an influence on animosity (Shoham et al., 2006), including nationalism, dogmatism together with internationalism. Additionally, nationalism, patriotism, and internationalism were proven to affect animosity (Al Ganideh and Elahee, 2018), and patriotism was demonstrated to positively impact economic animosity (Mainolfi, 2021). As an antecedent of animosity, “nationalism is commitment plus exclusion of others, a readiness to sacrifice bolstered by hostility toward others” (Druckman, 1994, p. 48); nationalism “encompasses views that one's country is superior and should be dominant (and thus implies a denigration of other nations)” (Balabanis et al., 2001, p.160). “Patriotism refers to strong feelings of attachment and loyalty to one's own country without the corresponding hostility towards other nations” (Balabanis et al., 2001, p.160). Ethnic identity, which represents individuals' identification with their ethnic groups, may also bear upon animosity. For the sake of enrichment of the literature on determinants of animosity, the effects of ethnic identity on animosity have been investigated in this study.

Ethnic feelings and pride can dictate relations between the in-group and the out-group (Hammond and Axelrod, 2006), and bring about animosity (Al Ganideh and Elahee, 2018). Ethnicity pertains to individuals' ascriptive social identity and is inherited, which cannot be altered by a person. Ethnicity, being a constant factor leads to conflict (Schlee, 2008), and as a consequence, animosity; thereby, ethnic identity is assumed as a significant source of animosity (Al Ganideh and Elahee, 2018). Besides, Korean people exhibit a unique ethnic identity, which may be derived from cultural values held by Koreans (Jun et al., 2021). One of the typical characteristics of Korean ethnic identity is the community spirit that places community interests above the needs of individuals (Lee and Park, 2017). When the interests of the Korean community are damaged, they may show negative feelings and even hostility to the offending country. Therefore, when Korean people are confronted with Japan's export restrictions to Korea, which cause harm to the interests of their own nation, they are inclined to show negative feelings and even animosity toward the offending country. The stronger the ethnic identity Koreans own, the more hostile feelings they tend to develop toward the offending country. Hence, in the research, ethnic identity may play a role in inflaming Korean people's economic animosity toward Japan in the context of Japan's export restrictions to Korea. Thus, the hypothesis was formulated as below to identify the bearing of ethnic identity upon economic animosity.

Hypothesis 1 (H1): Ethnic identity will negatively influence economic animosity.

### Media uses

As mass media and social media have become ubiquitous in the world, they grow increasingly significant in our daily life. The way mass media deal with social issues affects the diversity perception of members of society (Tichenor et al., 1980). It is said that the more people use traditional newspapers, the stronger the solidarity among members of society (Stamm, 1985). Ethnic identity could be a motivation to utilize ethnocentric news. When two countries are in conflict, people could be motivated. These processes can be explicated by way of the uses and gratification theory (Katz et al., 1973), as representative dimensions of uses and gratification are companionship, passing time, enjoyment, social interaction, escape, relaxation, etc. (Griffin et al., 2019). Another concept that describes media use as a more active user rather than a passive audience is the need for orientation (Valenzuela and McCombs, 2019). This study uses ethnic identity as an ethnic orientation and motivational element.

Additionally, explorations have revealed that ethnicity affects consumers' perspectives on products of nations associated with their ethnic origins (Little and Singh, 2014). In accordance with Jun et al. (2014), people with higher Korean ethnic identities consume more Korean (ethnic) TV shows, movies, and music. This is an example in point to prove the effects of ethnic identity on media content consumption. Besides, in the research of Jun (2022), it has been demonstrated that ethnic identity exerts a significant positive bearing upon the consumption of boycott media news and boycott SNS information. In other words, it has been found that Korean people with higher ethnic identities watch much more boycott news (Jun, 2022). Given this, ethnic identity's effects on the consumption of news on the boycott against Japan can be hypothesized as follows.

Hypothesis 2a (H2a): Ethnic identity will positively influence the use of boycott news.

News, as a primary source of enhancing social interaction and learning, has constantly been essential for individuals' daily life. At present, news can be easily obtained in people's lives, as people can gain news exposure *via* TV, mobile devices, desktops, and laptops. As for the influence of news exposure, it has been probed into since the commencement of mass communication research. News consumption directly generates an effect on individuals' perceptions and attitudes, which may result in follow-up actions (Namkoong et al., 2017). Besides, the findings of Bi et al. (2021) have manifested that Americans who consume more news about trade conflicts on social media are more inclined to alter their attitudes toward Chinese people against the background of the US–China trade conflict. Moreover, newspaper coverage engenders a significant influence on anger (Li et al., 2021).

News has impacts on a certain belief; this relationship can be explained by cultivation effects. Reality represented by media shows long-term and cumulative effects on users (Gerbner, 1969), and the relationship between TV and users' reality perception has been supported (Morgan and Shanahan, 1997). Cultivation theory has expanded its coverage from TV to video (Dobrow, 1990), rock music (Sherman and Dominick, 1986), cable TV (Cohen and Weimann, 2000), and online games (Williams, 2006). In addition, heavy use of local U.S. TV news affects viewers' negative responses toward African Americans (Arendt and Northup, 2015), which is a case in point to demonstrate the bearing of news on a certain belief; thus, boycott news may influence Korean individuals' certain belief about Japan.

Furthermore, in alignment with the outcomes of Kim and Kim (2020), news editorials can trigger animosity among consumers, as consumers tend to put their trust in information gained from news media, ultimately assenting to the viewpoints of the newspaper. Newspapers have been utilized for measurement of the extent of conflict (Du et al., 2017), as public opinions on issues with other foreign nations may be shaped *via* newspapers of their own country (Kim and Kim, 2020). Alvarez and Campo (2020) have investigated the causes of individuals' feelings of animosity, among which the topics about the most disliked countries in the news serve a vital part in leading to animosity. Consequently, the hypothesis was developed as follows to ascertain the effects of boycott news consumption on economic animosity.

Hypothesis 2b (H2b): The use of boycott news will positively influence economic animosity.

### Boycott intention

The word boycott originated in the 19th century when some small businessmen decided not to buy products provided by Charles Boycott (Cruz et al., 2013). Friedman (1985) has considered a boycott “an attempt by one or more parties to achieve certain objectives by urging individual consumers to refrain from making selected purchases in the marketplace” (pp. 97–98). Similar to a consumer or corporate boycott, a tourism boycott can be initiated collectively by individual persons for some reasons or represented by deputies as tools for international political negotiations (Castañeda and Burtner, 2010); nevertheless, it stands for forms of refusal to visit one or more specific tourism destinations, which is different from a consumer or corporate boycott (Castañeda and Burtner, 2010; Shaheer et al., 2019). Tourism boycott may engender huge and profound socioeconomic consequences, e.g., unfavorable tourist destination image along with the subsequent decrease in visitors (Shaheer et al., 2018).

Boycott intention indicates the willingness of consumers to take participation in a boycott in the future (Jeon, 2019). Besides, boycott intention has been deemed as the intention or willingness to conduct boycotts (Zhang et al., 2017). Boycott intention is highly restricted by animosity toward the boycott target (Palacios-Florencio et al., 2021). In other words, animosity is a determinant of boycott intention; that is, animosity produces a significant influence on boycott intention (Rose et al., 2009; Palacios-Florencio et al., 2021; Lee and Chon, 2022).

Prior research has explored associations between animosity and willingness/intentions of visiting certain destinations (Abraham et al., 2021). Study findings of Abraham et al. (2021) have denoted that animosity generates a negative bearing upon a willingness to visit a destination; specifically speaking, animosity toward the Chinese Government and Chinese nationals is negatively related to willingness to pay a visit to China. In alignment with the results of Stepchenkova et al. (2018), tourists' willingness and intention to pay a visit to some destinations can be affected by individuals' animosity toward that country. Accordingly, consumer animosity may reduce outbound tourists' tendencies to make choices of the offending countries as their tourist destinations (Alvarez and Campo, 2014; Sánchez et al., 2018). Furthermore, it has been demonstrated that consumer animosity engenders significant positive impacts on Korean consumers' intention to boycott Japanese food and clothes, as well as travel to Japan (Lee and Chon, 2022).

Hence, Japan's successive economic confrontation has led to a campaign to boycott Japanese products and the phenomenon of boycotting tourism to Japan, which may result from the economic animosity of Korean consumers triggered by Japan's export restrictions to Korea. As a consequence, in light of the aforementioned research, we posit that the economic animosity of Korean people will positively affect the boycott intention to visit Japan.

Hypothesis 3 (H3): Economic animosity will positively influence boycott visit intention.

### Government trust

Trust, a state of psychology, encompasses a willingness of accepting vulnerability due to positive expectations of other people's behaviors or intentions (Rousseau et al., 1998). Emotions play crucial roles in trust's generation, conservation, or deterioration (Jiménez and Martín, 2014); positive emotions are able to increase trust (Jiménez and Martín, 2010), whereas negative emotions can erode and reduce trust (Kiefer, 2005; Jiménez and Martín, 2010). Multiple scholars (e.g., Getha-Taylor, 2012; He and Ma, 2021) have made endeavors to comprehend trust in government to seek out means of restoring it *via* better public performance and management. According to Thomas (1998), three dimensions of trust in government have been identified, namely, fiduciary trust, mutual trust, along with social trust.

Up to the present, few attempts have been made to verify the bearings of animosity upon government trust, but the effects of animosity on trust have been investigated in several empirical studies, which have indicated that consumer animosity harbors considerable implications on consumer trust (Jiménez and Martín, 2010, 2014). To be more specific, in line with the research finding of Jiménez and Martín (2010, 2014), animosity negatively bears upon trust. Although arising from a particular event, animosity is a general emotion of hostility, manifested in mistrust and negation of everything that consumers deem as the representative of a certain country (Jiménez and Martín, 2014), comprising its government. Besides, since negative emotions can diminish trust (Kiefer, 2005; Jiménez and Martín, 2010), animosity, as an emotion of hostile feelings, may bring about a reduction in government trust. Accordingly, we expect that Korean individuals' economic animosity toward Japan will reduce their trust in the Japanese government. Thereby, the hypothesis was put forward to determine the relationship between economic animosity and Japanese government trust.

Hypothesis 4a (H4a): Economic animosity will negatively influence Japanese government trust.

Trust produces a series of beliefs together with positive expectations for the future behavior of all parties (Singh and Sirdeshmukh, 2000). Trust serves vital roles in building relationships among consumers and companies at that moment when they own diverse origins of society and culture (Dahlstrom and Nygaard, 1995). Besides this, under the frame of commitment-confidence theory, trust performs an essential part in prompting purchase intention (Singh and Sirdeshmukh, 2000). The research of Jiménez and Martín (2007) probed into the impacts of trust on purchase intention, and the findings have suggested that the trust of consumers has a positive bearing on consumers' purchase intention of foreign products. Consequently, trust is a motivating factor for purchase intention.

As a boycott is one party or more parties' attempt to avoid selected purchases for the sake of the attainment of the targets in the marketplace (Friedman, 1985), in this article, boycott intention signifies consumers' intention or willingness to make an attempt to refrain from making selected purchases to attain particular objectives. In accordance with the outcomes of Jiménez and Martín (2014), trust can enhance consumers' purchase intention of a certain foreign country's products; hence, the trust may reduce the consumers' intention of attempting to avoid the purchase of that country's products or boycott intention. Moreover, trust in the government is believed to be associated with the boycott, especially distrust in the government (Sato, 2022). Therefore, we anticipate that government trust will harbor a negative influence on boycott intention, and the hypothesis was established to identify associations between government trust and boycott intention.

Hypothesis 4b (H4b): Japanese government trust will negatively influence boycott visit intention.

### Research model

**Figure 1:**
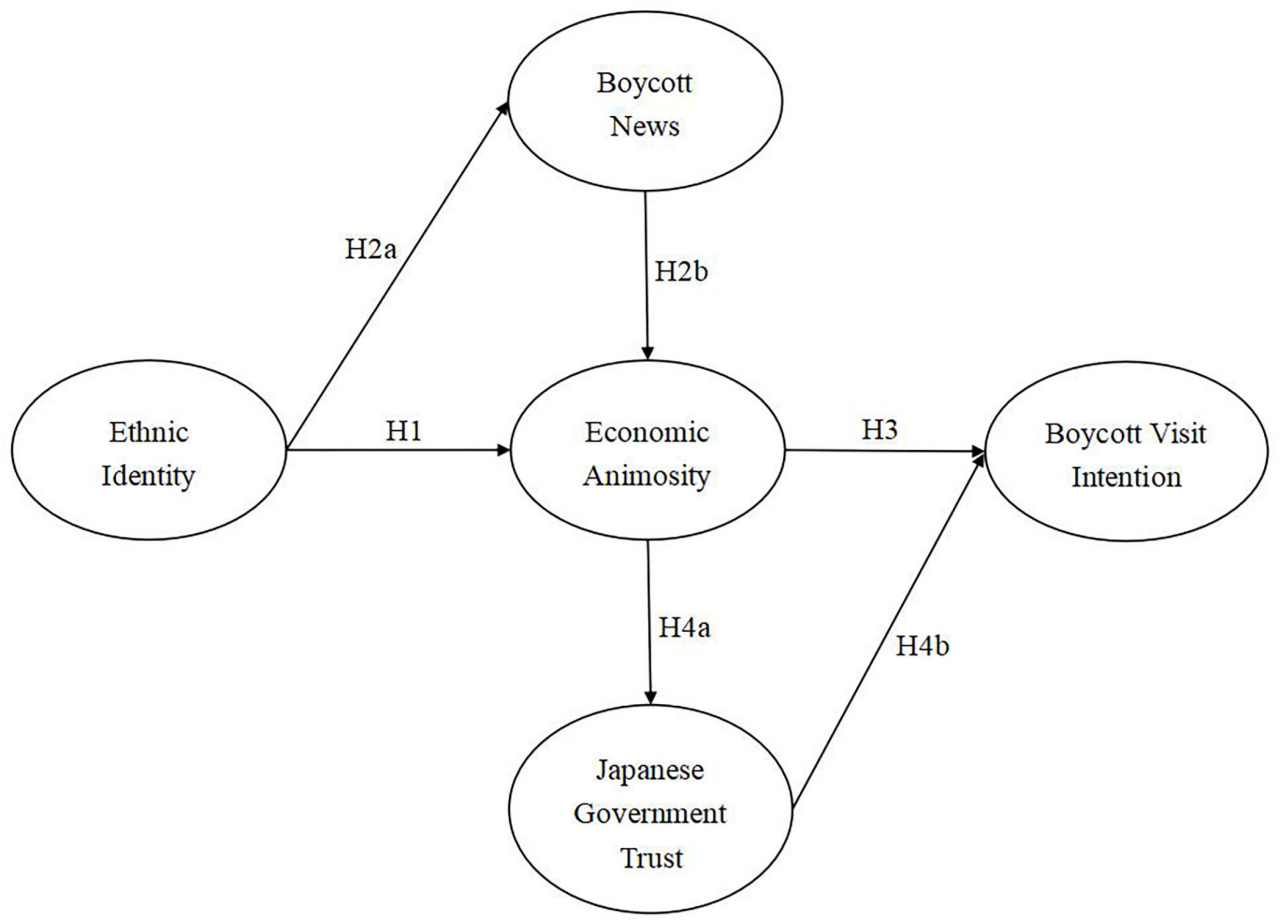
Research model.

In alignment with theoretical discussion as well as research hypotheses, we set up the following research model for the relations among all the variables (see Figure 1).

## Materials and methods

### Sample characteristics

This investigation adopted a survey research method for verification of the proposed model, and a professional research firm was recruited to collect general samples. Research panels from the research firm were requested to take participation in the survey *via* a web page established by the research firm. As a result, 333 samples were collected, with 333 Korean adult respondents having participated in the survey. Subsequently, the validity of the samples was judged to exclude the samples with insincere responses and missing values, and thereupon, the samples were collated for data analysis.

The frequency analysis of respondents' demographic characteristics was conducted (see Table 1). Concerning gender, 163 respondents (48.9%) are male, and 170 (51.1%) are female. According to the results of the descriptive statistics, the ages of respondents are between 20 and 72, with a mean age of 40.8. Specifically, 22.8% are in their 20s, 24.0% are 30–39 years old, 40–49 are 26.5%, and 26.7% are in their 50s and older. Regarding current residence, 27.3% are living in Seoul, 27.6% are in Gyeonggi-do, 6.6% are living in Busan and Incheon, respectively, and 31.9% are in other Korean regions. In addition, regarding occupation, 57.1% are office workers, 4.5% own their individual business, 9.9% work as professionals, 13.2% are housewives, 8.1% are students, and 7.2% have other occupations.

**Table 1:** Respondents' demographic characteristics.

**Variable**	**Item**	**Frequency**	**Percentage**
Gender	Male	163	48.9
	Female	170	51.1
Age	29 years old and below	76	22.8
	30–39 years old	80	24.0
	40–49 years old	88	26.5
	50 years old and above	89	26.7
Current residence	Seoul	91	27.3
	Gyeonggi-do	92	27.6
	Busan	22	6.6
	Incheon	22	6.6
	Other Korean regions	106	31.9
Occupation	Office worker	190	57.1
	Individual business	15	4.5
	Professional	33	9.9
	Housewife	44	13.2
	Student	27	8.1
	Other	24	7.2

### Measurements

In the research, measurement items for ethnic identity, economic animosity, Japanese government trust, and boycott visit intention were built in line with extant literature.

Considering ethnic identity, it was measured *via* seven items, which originated from Jun et al. (2014), covering “I'm very proud of my Korean ethnic background,” “I am highly attached to Korean culture,” “I love Korean culture and tradition,” “I am influenced by Korean history, tradition, customs, and ethics,” “I think in Korean way in many aspects of my life,” “I have a deep understanding of Korean culture,” and “I feel strong ethnic identification”.

In terms of boycott news use, it was assessed by means of three items, incorporating “I saw a lot of boycott articles in the newspaper,” “I saw a lot of boycott articles in the broadcasting,” as well as “I saw a lot of boycott articles online”.

As for economic animosity, five items were adapted from the article of Nakos and Hajidimitriou (2007), encompassing “Japan is not a reliable trading partner,” “Japan wants to gain economic power over Korea,” “Japan is taking advantage of Korea,” “Japan has too much economic influence in Korea” together with “The Japanese are doing business unfairly with Korea”.

Japanese government trust was conducted measurement through four items that were modeled after Chaudhuri and Holbrook (2001), consisting of “I trust the Japanese government,” “The Japanese government is believable,” “I can rely on the Japanese government” along with “The Japanese government delivers trustworthy information”.

Moreover, boycott visit intention was conducted an assessment through three items, which were developed based on the study of Stafford et al. (1996), including “Likely,” “Possible,” and “Probable”.

The aforementioned items were measured by using a 7-point Likert scale where one signifies strongly disagree and seven stands for strongly agree.

### Data analysis

Exploratory factor analysis (EFA for short) together with reliability analysis has been performed *via* SPSS for the sake of the inspection of factor structures and the measurement model's reliability. To be more concrete, factor loadings, communicalities, and eigenvalues were employed to check the factors' validity (Hair et al., 2009), and Cronbach's α was used for verification of the factors' reliability.

Confirmatory factor analysis (CFA for short) has been implemented using Amos for the sake of the verification of the construct validity. A number of measurement indices are utilized to appraise goodness-of-fit (Bentler and Bonett, 1980). In this study, the normalized χ^2^ (χ^2^/*df* or CMIN/DF), Comparative Fit Index (CFI), Incremental Fit Index (IFI), Tucker Lewis Index (TLI) together with Root Mean Square Error of Approximation (RMSEA) were employed. After ascertaining the measurement model's fit, convergent validity along with discriminant validity was inspected for evaluation of the model's construct validity. A standard of convergent validity is a calculation of composite reliability as well as each construct's average variance extracted (AVE) (Fornell and Larcker, 1981). Discriminant validity tests if one construct differs from others (Collier, 2020). Regarding the examination of discriminant validity, a comparison of a construct's square roots of AVE with correlation coefficients among constructs is requisite (Fornell and Larcker, 1981). Additionally, this article adopted a statistical method of SEM through Amos by way of the maximum likelihood estimation method to verify the hypotheses.

## Results

### Measurement model

The Kaiser–Meyer–Olkin (KMO) test and Bartlett's test of sphericity were adopted for evaluation of the original variables' suitability for EFA before EFA was conducted. The KMO test result lay at 0.896 (illustrated in Table 2), surpassing the acceptable level of 0.50 (Hair et al., 2009; Mooi et al., 2018), so it was demonstrated that original variables were desirable for EFA. Besides this, the significant probability was statistically significant (*p* < 0.001) through verification of Bartlett's test of sphericity, proving the sample data's suitability for EFA (Hair et al., 2009). Subsequently, EFA was implemented with principal component analysis being the extraction method and Varimax with Kaiser Normalization being the rotation method.

**Table 2:** KMO and Bartlett's test of sphericity results.

**KMO**	**Bartlett's test of sphericity**	
	**Approximate chi-square**	**Degree of freedom (*df*)**
0.896	7,410.889	231

The EFA results (see Table 3) reveal that from 22 original variables or items, five factors were extracted, among which boycott news, Japanese government trust, as well as boycott visit intention were named based on the hypotheses and the results of Rotated Component Matrix, and ethnic identity together with economic animosity was named in accordance with previous research (Nakos and Hajidimitriou, 2007; Jun et al., 2014) and the results of Rotated Component Matrix as well. Factor loadings of ethnic identity (EI), boycott news (BN), economic animosity (EA), Japanese government trust (JGT), and boycott visit intention (BVI) ranged between 0.714 and 0.931, over 0.50, denoting that all the items ought to be retained (Hair et al., 2009). Additionally, the range of the original variables' communicalities was between 0.591 and 0.972, surpassing 0.50, the recommended threshold (Mooi et al., 2018). Concretely, all of the communicalities were sufficiently high, so all the variables in this study had a sufficient explanation and could be reserved. Besides, the eigenvalues of the five extracted factors were 4.768, 1.988, 3.573, 3.958, and 2.775, so the minimum of the eigenvalues was 1.988, over 1, manifesting that each extracted factor occupied more variance than a single variable, satisfying the requirements of Kaiser criterion or K1, namely, eigenvalue-greater-than-one rule (Kaiser, 1960); accordingly, the extracted factors could be preserved. Plus, all the extracted factors' variance explanation power reached 77.553%, which lay well above the minimum requirement of 50% (Mooi et al., 2018) and indicated that the original variables' information loss amount was not much, proving the ideal effect of EFA.

**Table 3:** Results of EFA and reliability analysis.

**Factor**	**Item**	**Mean**	**SD**	**Factor loading**	**Communality**	**Eigen value**	**% of variance**	**Cronbach's α**
Ethnic identity (EI)	I'm very proud of my Korean ethnic background. (EI1)	5.08	1.325	0.795	0.663	4.768	21.675	0.919
	I am highly attached to Korean culture. (EI2)	5.07	1.188	0.807	0.697			
	I love Korean culture and tradition. (EI3)	5.15	1.275	0.823	0.732			
	I am influenced by Korean history, tradition, customs, and ethics. (EI4)	5.14	1.172	0.777	0.635			
	I think in Korean way in many aspects of my life. (EI5)	5.06	1.187	0.731	0.616			
	I have a deep understanding of Korean culture. (EI6)	4.77	1.187	0.831	0.721			
	I feel strong ethnic identification. (EI7)	5.08	1.221	0.885	0.811			
Boycott news (BN)	I saw a lot of boycott articles in the newspaper. (BN1)	4.53	1.551	0.753	0.605	1.988	9.036	0.730
	I saw a lot of boycott articles in the broadcasting. (BN2)	5.13	1.293	0.855	0.789			
	I saw a lot of boycott articles online. (BN3)	5.42	1.281	0.745	0.652			
Economic animosity (EA)	Japan is not a reliable trading partner. (EA1)	4.77	1.713	0.732	0.669	3.573	16.240	0.889
	Japan wants to gain economic power over Korea. (EA2)	5.34	1.603	0.746	0.797			
	Japan is taking advantage of Korea. (EA3)	5.55	1.461	0.714	0.753			
	Japan has too much economic influence in Korea. (EA4)	4.88	1.628	0.761	0.591			
	The Japanese are doing business unfairly with Korea. (EA5)	5.37	1.517	0.771	0.772			
Japanese government trust (JGT)	I trust the Japanese government. (JGT1)	2.02	1.403	0.881	0.873	3.958	17.990	0.969
	The Japanese government is believable. (JGT2)	1.88	1.303	0.920	0.936			
	I can rely on the Japanese government. (JGT3)	1.83	1.279	0.931	0.937			
	The Japanese government delivers trustworthy information. (JGT4)	1.80	1.257	0.907	0.908			
Boycott visit intention (BVI)	Likely (BVI1)	5.53	1.743	0.836	0.965	2.775	12.612	0.991
	Possible (BVI2)	5.55	1.763	0.837	0.969			
	Probable (BVI3)	5.55	1.758	0.854	0.972			
All factors							77.553	0.809

Reliability analysis was performed *via* SPSS, and Cronbach's α values for the factors (shown in Table 3) were 0.919, 0.730, 0.889, 0.969, and 0.991, over the reference value of 0.70 (Mooi et al., 2018), indicating the measurements' high reliability.

In addition, the measurement model's goodness of fit reached a satisfactory level (illustrated in Table 4); the value of CMIN/DF lay at 2.626, beneath the acceptable level of 3.0 (<3.0) (Kline, 2011). CFI, IFI along with TLI were 0.956, 0.956, as well as 0.949, respectively, outweighing the recommended acceptable value of 0.90 (>0.90) (Bentler and Bonett, 1980), and RMSEA was 0.070, comforting to the requirement (≤0.08) (MacCallum et al., 1996).

**Table 4:** Fitting test.

**Model fit index**	**Evaluation index**	**Model's values**
CMIN/DF	<3.0	2.626
CFI	>0.90	0.956
IFI	>0.90	0.956
TLI	>0.90	0.949
RMESA	≤0.08	0.070

Concerning the measurement of convergent validity, composite reliability values of EI, BN, EA, JGT, and BVI (shown in Table 5) were 0.920, 0.765, 0.896, 0.970, and 0.991, outstripping the acceptable level of 0.70 (Fornell and Larcker, 1981). Moreover, AVE values of EI, BN, EA, JGT, and BVI (see Table 5) were 0.621, 0.531, 0.638, 0.890, and 0.974, surpassing the threshold of 0.50 (Fornell and Larcker, 1981). Accordingly, the research model's convergent validity was demonstrated. Because all latent variables' square roots of AVE (illustrated in Table 6) were 0.788, 0.729, 0.799 0.943, and 0.987, exceeding correlation coefficients between variables, the research model's discriminant validity was demonstrated.

**Table 5:** Convergent validity analysis.

**Latent variable**	**Item**	**Std. estimate**	**Composite reliability**	**AVE**
EI	EI1	0.782	0.920	0.621
	EI2	0.765		
	EI3	0.794		
	EI4	0.734		
	EI5	0.720		
	EI6	0.812		
	EI7	0.898		
BN	BN1	0.574	0.765	0.531
	BN2	0.917		
	BN3	0.649		
EA	EA1	0.766	0.896	0.638
	EA2	0.902		
	EA3	0.864		
	EA4	0.567		
	EA5	0.850		
JGT	JGT1	0.904	0.970	0.890
	JGT2	0.962		
	JGT3	0.963		
	JGT4	0.944		
BVI	BVI1	0.984	0.991	0.974
	BVI2	0.987		
	BVI3	0.989		

**Table 6:** Discriminant validity analysis.

**Latent variable**	**EI**	**BN**	**EA**	**JGT**	**BVI**
EI	0.788				
BN	0.387	0.729			
EA	0.249	0.211	0.799		
JGT	−0.138	−0.148	−0.574	0.943	
BVI	0.235	0.188	0.763	−0.542	0.987

### Structural model

Evaluating the proposed research model's goodness-of-fit is the initial step of SEM analysis. In the exploration, CMIN/DF was found to be 2.584 (524.499/203), which was lower than the acceptable level of 3.0 (<3.0) (Kline, 2011). CFI, IFI, together with TLI were 0.956, 0.957, and 0.950, respectively, surpassing the reference value (>0.90) (Bentler and Bonett, 1980), and RMSEA was 0.069, below the recommended threshold (≤0.08) (MacCallum et al., 1996). Considering the measures, this model's goodness-of-fit is considered acceptable. For enhancement of the model's goodness-of-fit, modification indices were utilized for ascertainment of any theoretically meaningful paths/relations, which were not encompassed in the original model. It was revealed that four sets of observed variables harbored covariance among the same constructs. Thereby, a second analysis was done for the modified model, and the findings manifested the revised model could fit data better than the original model, as CMIN/DF was 1.777, CFI, IFI, as well as TLI were 0.979, 0.979 along with 0.976, and RMSEA was equal to 0.048 (see Table 7).

**Table 7:** Final fitting test.

**Model fit index**	**Evaluation index**	**Model's values**
CMIN/DF	<3.0	1.777
CFI	>0.90	0.979
IFI	>0.90	0.979
TLI	>0.90	0.976
RMESA	≤0.08	0.048

**Figure 2:**
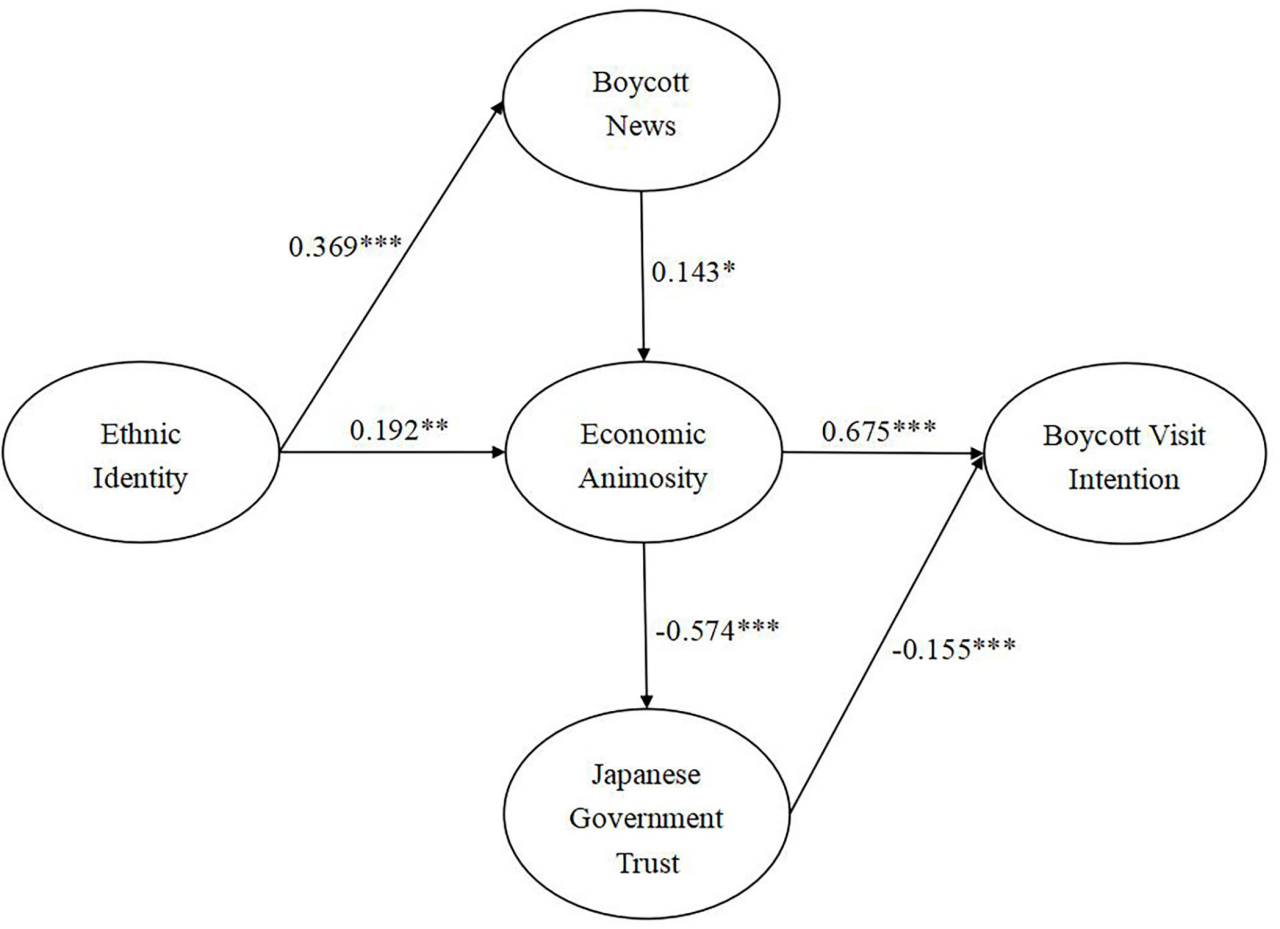
Hypotheses testing results. The *, **, and *** symbols indicate the values of *p < 0.05, **p < 0.01, and ***p < 0.001 respectively.

All hypotheses were tested, and the revised model suggested all six hypotheses were accepted (see Table 8 and Figure 2). Concerning H1, ethnic identity exerted significant direct impacts upon economic animosity (H1: β = 0.192, *p* < 0.01), and mediating effects of boycott news were proved as well. Ethnic identity positively affected boycott news (H2a: β = 0.369, *p* < 0.001), and boycott news was linked to economic animosity (H2b: β = 0.143, *p* < 0.05). Thus, this article demonstrated the mediating role of boycott news with direct relations of ethnic identity to economic animosity. Regarding the consequences of economic animosity, it significantly positively affected boycott visit intention (H3: β = 0.675, *p* < 0.001). It was evinced that the Japanese government trust mediated between economic animosity and boycott visit intention. Concretely speaking, economic animosity negatively bore upon Japanese government trust (H4a: β = −0.574, *p* < 0.001), and Japanese government trust negatively impacted boycott visit intention (H4b: β = −0.155, *p* < 0.001).

**Table 8:** SEM test results.

**Hypotheses**	**Paths**	**β**	**C.R**.	***p***	**Conclusion**
H1	EI→ EA	0.192	3.088	0.002	Supported
H2a	EI→ BN	0.369	5.381	***	Supported
H2b	BN→ EA	0.143	2.183	0.029	Supported
H3	EA→ BVI	0.675	12.713	***	Supported
H4a	EA→ JGT	−0.574	−10.831	***	Supported
H4b	JGT→ BVI	−0.155	−3.299	***	Supported

*** p < 0.001.

## Conclusion and discussion

### Conclusion

This article investigated influencing factors of boycott intention to visit Japan with economic animosity being a main mediating variable. Ethnic identity and boycott news use serve as antecedents of economic animosity; Japanese government trust and boycott visit intention function as consequences of economic animosity. In accordance with the empirical analysis results, ethnic identity engenders a significant direct positive bearing on economic animosity and boycott news, boycott news significantly positively affects economic animosity; thereby boycott news use's mediating role between ethnic identity and economic animosity has been proved. In terms of consequences of economic animosity, economic animosity significantly negatively impacts Japanese government trust and positively influences boycott visit intention, Japanese government trust significantly negatively affects boycott visit intention, and Japanese government trust mediates between economic animosity and boycott intention to visit Japan.

First, H1 has been supported, as the study findings suggest that ethnic identity significantly positively bears upon economic animosity, which is in alignment with Al Ganideh and Elahee (2018), in which it has been assumed that ethnic identity is a crucial source of animosity. As ethnic identity, identification with an ethnic group is considered to be individual behavior's point of reference, and also impacts individuals' unfavorable feelings toward certain foreign countries (El Banna et al., 2018), for instance, animosity. Hence, it has been demonstrated that Korean consumers' ethnic identity engenders a significant direct positive influence on economic animosity toward Japan. In other words, Korean consumers with higher ethnic identities may harbor more economic animosity toward Japan.

Second, the research outcomes denote that ethnic identity significantly positively impacts boycott news use, so H2a has been accepted. According to prior research by Jun (2022), ethnic identity generates significant positive effects on the consumption of boycott media news along with boycott SNS information; to be more specific, Korean people with higher ethnic identity watch or listen to more boycott news. Furthermore, it has been revealed that immigrants from Korea with higher ethnic identity consume more ethnic media content, such as TV shows, movies, and music (Jun et al., 2014), which is a case in point to reveal the positive influence of ethnic identity on media content use. Thus, the more ethnic identity Korean people have, the more boycott news they use.

Third, H2b has been adopted, as the study results manifest that news about the boycott of Japan significantly positively affects economic animosity. News consumption directly produces an effect on people's perceptions and attitudes, which may bring about follow-up actions (Namkoong et al., 2017). Multiple prior investigations have demonstrated the effects of news on animosity (Alvarez and Campo, 2020; Kim and Kim, 2020). In line with the outcomes of Kim and Kim (2020), news editorials can give rise to consumers' animosity. The findings of Alvarez and Campo (2020) have denoted that the topics about the most disliked countries in the news perform a vital role in triggering feelings of animosity. Therefore, the more news about boycotting Japan Korean consumers use, the more economic animosity toward Japan they harbor. In addition, as H2a and H2b have been supported, boycott news mediates between ethnic identity and economic animosity. To be more specific, the exploration proves mediating effects of boycott news with direct relations of ethnic identity to economic animosity.

Fourth, in alignment with the study results, economic animosity significantly positively influences boycott visit intention, so H3 has been accepted. This research finding is in line with previous explorations (Rose et al., 2009; Palacios-Florencio et al., 2021), in which it has been revealed that animosity generates a significant impact on boycott intention, namely, animosity is a motivating factor for boycott intention. Moreover, it has been demonstrated that consumer animosity significantly positively affects Korean consumers' intention of boycotting Japanese food and clothes together with travel to Japan (Lee and Chon, 2022), which is also a supporting point for the positive bearings of animosity on boycott intention. Consequently, as the result shows, economic animosity serves as a predictor of boycott visit intention.

Fifth, it has been found that economic animosity significantly negatively impacts Japanese government trust, so H4a has been adopted. Emotions play an important part in trust's generation, conservation, or deterioration (Jiménez and Martín, 2014); positive emotions can raise trust (Jiménez and Martín, 2010), while negative emotions can decrease trust (Dunn and Schweitzer, 2005; Kiefer, 2005; Jiménez and Martín, 2010). Animosity, an emotional reaction, has a negative bearing on consumer trust (Jiménez and Martín, 2010, 2014). Thus, although arising from some particular event, animosity is a general emotion of hostility, manifested in mistrust along with negation of everything that consumers consider as the representative of that country (Jiménez and Martín, 2014), encompassing the certain country's government. As a consequence, the economic animosity of Korean consumers attenuates their trust in the Japanese government.

Sixth, the research has supported H4b, as it has been manifested that Japanese government trust generates a negative effect upon boycott visit intention. In accordance with the research of Jiménez and Martín (2014), trust promotes consumers' purchase intention of a certain foreign country's products. Thereby, trust may reduce consumers' boycott intention, namely, the intention of attempting to avoid the purchase of a certain foreign country's products. Besides, trust in the government is considered to be connected to boycotts, particularly distrust in the government (Sato, 2022). Therefore, the more trust in the Japanese government Korean consumers have, the lower the boycott intention they harbor. Moreover, as H4a and H4b have been accepted, it has been proved that Japanese government trust performs a mediating role between economic animosity and boycott visit intention.

### Implications

#### Theoretical implications

Multiple academic implications have been furnished in this article. First, the investigation theoretically articulates and empirically demonstrates the significant positive impacts of ethnic identity and boycott news use on economic animosity; thereby, the antecedents of economic animosity have been empirically supported and proved. Although determinants of animosity have been explored in prior research (Mrad et al., 2013; Kim and Kim, 2020), there has hardly been any attempt to ascertain the effects of ethnic identity or boycott news upon economic animosity. This study supplies supporting evidence for the significant positive influence of ethnic identity and boycott news on economic animosity. Besides, the article proves the mediating role of boycott news use between ethnic identity and economic animosity, which has been ignored in extant research.

Next, the study findings have held up the significant positive bearing of ethnic identity on boycott news use, which is consistent with the outcomes of Jun (2022), which have denoted that ethnic identity significantly positively affects the consumption of boycott media news as well as boycott SNS information. Namely, the stronger ethnic identity Koreans harbor, the more boycott news they watch or listen to. Thus, the exploration replenishes the literature on the positive effects of ethnic identity upon boycott news use.

In addition, the research has probed into factors that impact boycott intention to visit Japan and demonstrated the positive bearing of economic animosity on boycott visit intention, and the negative impact of Japanese government trust on boycott visit intention. Although Abraham et al. (2021), and Stepchenkova et al. (2018) examined the bearings of animosity on visit intention to a destination, the impacts of animosity on boycott visit intention received scant attention. Hence, this investigation has not only filled the research gap but also empirically proved the positive influence of economic animosity on boycott visit intention.

Although trust has been defined across many disciplines, the relations between government trust and animosity have been neglected virtually. The study makes contributions to providing empirical support for associations between economic animosity and government trust. According to the research results, Korean consumers who harbor more economic animosity toward Japan exhibit distrust or less trust in the Japanese government. In addition, the article furnishes supporting evidence for the influencing factors of boycott visit intention (i.e., economic animosity, and Japanese government trust). Furthermore, this study is conducive to the exploration of the mediating role of government trust between economic animosity and boycott visit intention, which has rarely been investigated in extant research; therefore, this article offers a new perspective for government trust studies.

#### Managerial implications

In the study, a number of managerial implications have been provided as well. To begin with, it is worth noting that our research has raised awareness that animosity can impact potential tourists' boycott intention to visit a destination. To be more specific, the results have confirmed the fact that Korean people's economic animosity toward Japan significantly positively influences their boycott intention to visit Japan, which may directly hurt the Japanese tourism industry. Thus, the conclusions from this article are associated with the tourism industry. Besides this, as prior research demonstrated the negative bearing of animosity on visit intention (Abraham et al., 2021); hence, animosity functions as a useful factor in tourism research and the tourism industry. To alleviate the boycott visit intention of Korean individuals, it is a good way to attenuate their economic animosity. Thus, tourism stakeholders, along with destination marketers, especially tourism practitioners should monitor and pay close attention to international tourist–consumers' sentiments and maintain vigilance against rises in animosity of international tourist-consumers (Yu et al., 2020), and take necessary marketing measures to attempt to mitigate negative influences of animosity.

Additionally, the tourist industry is generally susceptible to diplomatic as well as political circumstances (Moufakkir, 2010); thus, government policymakers ought to be cognizant of the vulnerability of the tourist industry and develop a number of safeguards to prevent and lessen damage at the moment when international boycotts take place. Concretely speaking, large-scale boycotts may greatly impact the image of a country and influence all the parties involved in the tourism industry, so government policymakers ought to take contingency plans into consideration and prepare multiple contingency plans in advance to deal with massive boycotts.

Besides, it has been shown that government trust significantly negatively influences boycott visit intention, so the increase of trust in the Japanese government is conducive to mitigating Korean individuals' boycott visit intention. Government policymakers ought to pay attention to international tourist-consumers' trust in their own national government, and endeavor to formulate policies for the sake of the uplift of international tourist-consumers' trust in their own national government to alleviate potential international tourist–consumers' boycott intention to visit their country.

As it has been proved that boycott news significantly positively influences economic animosity, it is beneficial to organize special teams to observe and monitor boycott news in the targeting major source markets and build up crisis management teams to respond to the negative emotions timely and quickly for the sake of avoidance of negative emotions transforming into animosity toward their country. Also, social media posts ought to be frequently updated so as to offer the latest information to the public (Hall and Page, 2016), especially the news on the friendly diplomacy between the boycotted country and the boycotting country.

### Limitations and future research

Despite the numerous theoretical and managerial implications the article has offered, there are still multiple limitations. First, animosity is multidimensional (Amine et al., 2005), and six ones have been summarized (Yu et al., 2020), encompassing war, economic, political, religious, cultural, and people-related/social animosity. However, in the article, animosity is confined to economic animosity. Although it has been demonstrated that economic animosity triggered by Japan's export restrictions to Korea serves as a determining factor for the boycott intention of visiting Japan, other animosity dimensions may also perform a role in boycott intention. Thus, future research ought to cover other animosity dimensions, for instance, war animosity, political animosity, religious animosity, etc. To be more concrete, the determinants and consequences of war animosity, political animosity, religious animosity, etc. ought to be investigated in subsequent studies.

Although the data were collected during the period of ongoing animosity of Korean people toward Japan, economic animosity belongs to situational animosity in essence (Jung et al., 2002; Mrad et al., 2013) rather than stable one; thereby, follow-up research ought to be conducted to collect similar latest data to confirm whether economic animosity toward Japan lasts and if identical relations between economic animosity and boycott visit intention exist. Plus, the article only explored the associations between economic animosity and boycott visit intention of Korean people toward Japan; hence, succeeding research on the relations between economic animosity and boycott visit intention among other countries should be conducted. Finally, more potential antecedents and consequences of economic animosity toward a foreign country ought to be delved into in future investigations. For instance, the effects of nationalism, patriotism, and internationalism on economic animosity, along with the impacts of economic animosity on boycott purchase intention can be probed in the following studies.

## Data availability statement

The original contributions presented in the study are included in the article/supplementary material, further inquiries can be directed to the corresponding author/s.

## Ethics statement

Ethical review and approval was not required for the study on human participants in accordance with the local legislation and institutional requirements. The patients/participants provided their written informed consent to participate in this study.

## Author contributions

J-WJ was responsible for collecting data. LS and J-WJ were responsible for analyzing data, paper drafting, and paper editing. All authors contributed to the article and approved the submitted version.

## Conflict of interest

The authors declare that the research was conducted in the absence of any commercial or financial relationships that could be construed as a potential conflict of interest.

## Publisher's note

All claims expressed in this article are solely those of the authors and do not necessarily represent those of their affiliated organizations, or those of the publisher, the editors and the reviewers. Any product that may be evaluated in this article, or claim that may be made by its manufacturer, is not guaranteed or endorsed by the publisher.
